# Inhibition of TGFβ1 accelerates regeneration of fibrotic rat liver elicited by a novel two-staged hepatectomy

**DOI:** 10.7150/thno.52102

**Published:** 2021-03-04

**Authors:** Bo Zhang, Fanzheng Meng, Yao Liu, Yubin Yuan, Jizhou Wang, Dehai Wu, Yifeng Cui, Shugeng Zhang, Hongrui Guo, Shuhang Liang, Wei Wang, Matthew Klos, Sherry Morgenstern, Yufeng Liu, Linmao Sun, Kun Ma, Xirui Liu, Yan Wang, Jihua Han, Guangchao Yang, Chenyang Zheng, Xianying Li, Shuo Zhou, Changyong Ji, Qingquan Bai, Jiabei Wang, Lianxin Liu

**Affiliations:** 1 Department of General Surgery, Key Laboratory of Hepatosplenic Surgery, Ministry of Education, The First Affiliated Hospital of Harbin Medical University, Harbin 150000, China.; 2 Department of Hepatobiliary Surgery, Anhui Province Key Laboratory of Hepatopancreatobiliary Surgery, The First Affiliated Hospital of USTC, Division of Life Sciences and Medicine, University of Science and Technology of China, Hefei 230001, China.; 3 Department of General Surgery, Heze Municipal Hospital, Heze 274000, China.; 4 Department of Medical Oncology, The First Affiliated Hospital of USTC, Division of Life Sciences and Medicine, University of Science and Technology of China, Hefei, Anhui, 230001, P.R. China.; 5 Pediatric Cardiac and Thoracic Surgery, University Hospitals Cleveland Medical Center, Cleveland, OH 44106, USA.; 6 Department of General Surgery, The Third Affiliated Hospital of Harbin Medical University, Harbin 150000, China.; 7 Division of Life Sciences and Medicine, University of Science and Technology of China, Hefei 230001, China.

**Keywords:** ALPPS, future liver remnant (FLR), fibrosis, hepatic stellate cells (HSCs), LY2157299 (galunisertib)

## Abstract

**Aims:** Emerging evidence is demonstrating that rapid regeneration of remnant liver elicited by associating liver partition and portal vein ligation for staged hepatectomy (ALPPS) may be attenuated in fibrotic livers. However, the molecular mechanisms responsible for this process are largely unknown. It is widely acknowledged that the TGFβ1 signaling axis plays a major role in liver fibrosis. Therefore, the aims of this study were to elucidate the underlying mechanism of liver regeneration during ALPPS with or without fibrosis, specifically focusing on TGFβ1 signaling.

**Approach:** ALPPS was performed in rat models with *N*-diethylnitrosamine-induced liver fibrosis and no fibrosis. Functional liver remnant regeneration and expression of TGFβ1 were analyzed during the ALPPS procedures. Adeno-associated virus-shTGFβ1 and the small molecule inhibitor LY2157299 (galunisertib) were used separately or in combination to inhibit TGFβ1 signaling in fibrotic rats.

**Results:** Liver regeneration following ALPPS was lower in fibrotic rats than non-fibrotic rats. TGFβ1 was a key mediator of postoperative regeneration in fibrotic liver. Interestingly, AAV-shTGFβ1 accelerated the regeneration of fibrotic functional liver remnant and improved fibrosis, while LY2157299 only enhanced liver regeneration. Moreover, combination treatment elicited a stronger effect.

**Conclusions:** Inhibition of TGFβ1 accelerated regeneration of fibrotic liver, ameliorated liver fibrosis, and improved liver function following ALPPS. Therefore, TGFβ1 is a promising therapeutic target in ALPPS to improve fibrotic liver reserve function and prognosis.

## Introduction

The liver is a complex organ that performs a variety of metabolic, detoxification, and immune system functions. It is also an organ with strong regenerative capacities. Not only can the liver continue to perform complex physiological functions even after 70% of its mass has been resected surgically, it also has the capability to return to its original size. Regeneration of the liver involves the coordinated expansion of cholangiocytes, endothelial cells, hepatocytes, hepatic sinusoidal cells, hepatic stellate cells (HSCs), Kupffer cells, and portal vein fibroblasts 1. Because of this phenomenon, besides liver transplant, liver resection is the best option to cure liver cancer.

Two-staged hepatectomy including portal vein embolization (PVE) or portal vein ligation (PVL) remains part of the standard treatment of liver cancer. First, a branch of the portal vein supplying the tumor-bearing lobe is blocked (Step I). This results in growth of the contralateral functional liver remnant (FLR). After the FLR reaches a sufficient volume, the ischemic lobes are removed (Step II). The primary limitation of this approach is the long interval between the procedures (at least 2 months for PVE and four weeks for PVL), which allows tumor progression to occur 2-4. Further, if liver function of the FLR is insufficient, or adequate growth of the FLR does not occur, liver transplant may be the only viable option 4.

Associating liver partition and portal vein ligation for staged hepatectomy (ALPPS) is a novel two-staged hepatectomy. Like conventional two-staged hepatectomy, the procedure occurs in two steps with an especially important variation: partitioning of liver parenchyma along the falciform ligament. This results in accelerated growth of the FLR, allowing Step II to occur sooner, sometimes in as little as one week after Step I 5. Further, the deportalized liver that is to be resected still provides partial function, which helps the liver maintain its function until an adequate FLR size is reached 6, 7. Because of this unique advantage, ALPPS has been applied in patients whose FLR is insufficient, especially patients with multiple liver metastases derived from colon cancer. There are only a few reports on the application of ALPPS in the treatment of primary liver tumor, especially hepatocellular carcinoma with fibrosis. The main factor limiting the use of ALPPS in patients with liver fibrosis is the high mortality rate caused by insufficient regeneration and severe complications. The question of how well this new approach works in patients with liver fibrosis has not been answered. To date, results from some studies have shown that application of ALPPS in patients with hepatitis-related hepatocellular carcinoma, most of whom suffer from liver fibrosis or cirrhosis, possibly benefits patients 8, 9. Regardless, the rates of morbidity and mortality associated with the procedure were still a serious problem. While better selection of patients and variations in the technique could improve outcomes, the molecular mechanisms behind why ALPPS works or fails are largely unknown.

Few animal studies have been reported on the molecular mechanisms responsible for ALPPS-associated regeneration of fibrotic liver. However, the molecular mechanisms responsible for liver fibrosis have been well studied. Liver fibrosis is largely controlled by the transforming growth factor β (TGFβ) pathway 10, 11. TGFβ is part of the transforming growth factor superfamily, which primarily regulates immune cell and stem cell proliferation and differentiation. TGFβ activates HSCs, thereby inducing them to undergo epithelial-mesenchymal transition (EMT). Increased EMT-associated signaling results in altered extracellular matrix deposition 12, 13. TGFβ1 is produced as a precursor that undergoes post-translational cleavage, glycosylation, and hydrolysis before becoming a mature endocrine molecule. It has been shown that TGFβ1 can inhibit liver regeneration via mothers against decapentaplegic homolog (SMAD)2/3/4 proteins. Further, TGFβ1 signaling can alter cyclin expression in hepatocytes, thus stopping the cells from undergoing G1/S-phase transition and preventing regeneration 12-14.

Given the previously described role of TGFβ1 in liver fibrosis, we hypothesized that the TGFβ1 signaling pathway attenuates hepatocyte regeneration during ALPPS in fibrotic liver. This study describes an underlying mechanism of ALPPS-associated hepatocyte regeneration in fibrotic and non-fibrotic livers.

## Methods

### Animals

All animals received humane care according to the “Guide for the Care and Use of Laboratory Animals” and all experimental procedures were conducted under the supervision of the Animal Ethics Committee of Harbin Medical University (HMU), China (No. 2019033). Male Sprague-Dawley rats, 4 weeks of age, were purchased from the Experimental Animal Center of HMU. After arrival, the animals were given ad libitum access to food and water in a vivarium that was maintained at 23 ± 1 °C with a 12/12 h day/night cycle. Animals were randomly divided into groups, with 3-5 animals sacrificed for each data point.

### N-diethylnitrosamine (DEN) and carbon tetrachloride (CCL4) administration

DEN and CCL_4_ were used to induce liver fibrosis in rats separately. DEN (Sigma Aldrich, 73861, USA) was diluted in sterile phosphate-buffered saline (PBS) and intraperitoneally injected into rats twice a week at a dose of 25 mg/kg from the age of 6 weeks. Sterile PBS was injected into the control group. CCl_4_-treated rats were intraperitoneally injected with 40% CCl_4_ olive oil solution (v/v) at 1 ml/kg body weight twice a week. The DEN treatment and CCL_4_ treatment lasted up to 14 weeks. METAVIR scoring was used to evaluate the degree of liver fibrosis 15.

### Adeno-associated virus (AAV) vectors

AAV9 GFAP vectors designed to specifically transfect HSCs *in vivo* were generated by Genechem Co. LTD (Shanghai, China). A short hairpin RNA sequence targeting the *TGFβ1* gene was cloned and packaged into the AAV vector (CV258, GFAP-EGFP-MIR155). The vector also encoded a green fluorescent protein (GFP) reporter, allowing for cellular visualization. The TGFβ1 RNA interference sequences used were 5'ACCGCTAGCTAACTGGAGGCTTGCTGAAGGCTGTATGCTG and 3'CAGGACACAAGGCCTGTTACTAGCACTCACATGGAACAAATGGCCCAAGCTTGGT. TTCTCCGAACGTGTCACGT was used as control.

### LY2157299 (Galunisertib) administration

LY2157299, a TGFβR1-specific inhibitor, was obtained from Selleck (s2230, USA) and dissolved in 1% sodium carboxymethyl cellulose (CMC) (Selleck, s6703, USA). DEN-treated rats were given either 150 mg/kg galunisertib or 1% CMC intragastrically twice per day for a total of 7 days before the rats were sacrificed 16, 17.

### AAV delivery into rat livers

Rats were anesthetized by intraperitoneal injection of 2% pentobarbital. A longitudinal incision was then made in the abdomen to expose the portal vein. 8 × 10^11^ viral particles of AAV9-GFAP-shTGFβ1 or AAV9-GFAP-shNC in a final volume of 2 mL were injected into the portal vein with a 25-gauge needle, as described previously 18. Four weeks after AAV infection, ALPPS was performed.

### Development of the ALPPS rat model

After 4-5 days of DEN/PBS treatment (week 12), rats were weighed and anesthetized by intraperitoneal injection of 2% pentobarbital. A state of deep anesthesia was confirmed by the toe-pinch reflex. Step I: To simulate human ALPPS in rats, portal vein branches were ligated and liver parenchyma was split between the right and left middle lobes. The left lateral lobe (LLL) was resected first, as is often done clinically to remove small colonic liver metastases. Portal branches of the right lobe (RL) and right middle lobes (RML) were individually ligated with 5-0 silk. The portal vein branches and the hepatic artery of the left middle lobe (LML) as well as the caudate lobe (CL), which served as the FLR and represented 26-30% of the total liver volume, were conserved. The liver parenchyma was split between the deportalized RML and the normally perfused LML with bipolar forceps. Step II: Rats underwent a relaparotomy 48 h after Step I to remove the deportalized RL and RML.

### Liver sample acquisition

Blood was obtained from the intrahepatic vena cava before organ harvesting and centrifuged at 3000 ×*g* for 30 min. Serum aliquots were collected and stored at -80 °C. Serum alanine aminotransferase (ALT), albumin (ALB), and total bilirubin (TBIL) levels were determined using an automated chemical analyzer (Johnson Vitros 5600 automatic biochemical analyzer, USA). Liver tissues were washed repeatedly with precooled PBS, weighed, and aliquoted. Part of the liver tissue was snap frozen directly and preserved at -80 °C, and part of the tissue later used to extract RNA was placed in a protectant tissue reagent (Qiagen, 76104, Germany) for 24 h at room temperature and then stored at -80 °C. The remaining liver tissue was fixed in 10% formalin.

### Liver regeneration

Liver regeneration was assessed by calculating the ratio of total FLR, including LML and CL, to body weight (FLR/BW). 3-5 animals were used for each time point.

### Isolation and culture of primary hepatocytes (PHCs) and HSCs

A two-step collagenase perfusion method was used to isolate and purify rat hepatocytes (19). Briefly, FLR dissected from DEN-treated rats 48 h after ALPPS were perfused with calcium-free Hanks balanced salt solution (HBSS) and then perfused with HBSS containing calcium and collagenase type IV (Gibco, 17104019). Livers were minced and filtered through cotton gauze to liberate the hepatocytes. The hepatocytes were suspended in 7 mL of Earle's minimum essential medium with 10% fetal bovine serum (FBS), 2% penicillin/streptomycin, 1% glutamine, and 1% nonessential amino acids. The hepatocytes were purified from nonparenchymal cells and nonviable hepatocytes by Percoll density gradient centrifugation at 1000 g for 10 min at 4 °C.

HSCs were isolated from the FLR of DEN-treated or PBS-treated rats 48 h after ALPPS by enzymatic digestion of the liver with collagenase type IV (Gibco, 17104019), Pronase (Roche, 10165921001), and DNase (Sigma, DN25) followed by centrifugation of the crude cell suspension through a density gradient medium (Nycodenz) 20. 6-8 × 10^6^ cells from each rat FLR were cultured in Dulbecco's Modified Eagle Media (DMEM) with 10% FBS, 2% penicillin/streptomycin, and 1% glutamine. HSCs and hepatocytes were cultured at 37 °C in a humidified incubator with 5% CO_2_.

### BRL-3A culture

The rat hepatic cell line BRL-3A was obtained from National Collection of Authenticated Cell Cultures (Shanghai, China). BRL-3A was cultured in DMEM supplemented with 1% glutamine, 10% FBS, and 1% penicillin/streptomycin at 37 °C in a humidified incubator with 5% CO_2_.

### Co-culture

PHCs/BRL-3A cells were plated on 10 cm dishes (2 × 10^5^ cells/mL) and incubated at 37 °C in 5% CO_2_ for 48-72 h. When the cultures reached ~50% confluence, the medium was replaced with medium used to culture HSCs for 48 h. 10 ng/mL cytokine rat TGFβ1 (rTGFβ1) (Novoprotein, Shanghai, China) and 10 μM galunisertib dissolved in DMSO were added to the medium and the cells were incubated for 24 h.

### 5-Ethynyl-20-deoxyuridine (EdU) assay

Cells were incubated with EdU (Ribobio, Shanghai, China) for 2 h and processed according to the manufacturer's instructions. After washing with PBS, the cells were treated with 300 μL of Apollo reaction cocktail for 30 min. Then, the DNA contents of the cells in each well were stained with Hoechst. *In vivo*, 5 mg/kg EdU was injected intraperitoneally 24 h before the animals were sacrificed. The tissues were fixed in 10% neutral formalin, dehydrated, embedded in paraffin, and sectioned at 4 μm. The sections were incubated with Apollo reagent for 60 min and the nuclei were stained with Hoechst. The cells and tissue sections were visualized under a fluorescence microscope. Quantification of EdU-positive hepatocytes was performed by blinded manual counting of five random visual fields (200×).

### Cell Counting Kit-8 (CCK8) assay

CCK-8 assay was used to measure cell viability. Exponentially growing hepatocytes (100 μL, 1 × 10^5^ cells/mL) were seeded on 96-well plates. The plates were then incubated at 37 °C and 5% CO_2_ for 48 h. Subsequently, 10 μL of CCK-8 (Beyotime, China) was added to each well. After 2 h, the absorbance at 450 nm was measured using a spectrophotometer.

### Quantitative real-time PCR (qRT-PCR)

Total RNA was extracted from 50 mg of tissue using GeneJET RNA Purification Kit (Thermo Scientific, k0732, USA). RNA quality and quantity were assessed using a spectrophotometer. After generation of the complementary DNA sequence (Takara reverse transcription system, rr036, China), qRT-PCR amplification and data analysis were performed on an ABI StepOne Plus Biosystem System using TB Green Premix Ex Taq™ II (Takara, rr820, China). The primers used for qRT-PCR are listed in Supplementary Table 1.

### Protein analysis

Total protein was extracted from 50 mg of liver tissue using Tissue Extraction Reagent II (Invitrogen, fnn0081, USA) and an EDTA-free protease inhibitor cocktail kit (Roche, 04693132001). Protein concentration was determined using a BCA protein assay kit (Sangon biotech, c503021, Shanghai, China). Total protein was separated by acrylamide gel and transferred to nitrocellulose. After blocking with 5% bovine serum albumin and incubation with primary and secondary antibodies, blots were displayed on the film and quantified using ImageJ v1.53a (Wayne Rasband, National Institutes of Health, USA). The concentration of TGFβ1 in each sample was measured using an ELISA kit (Sangon biotech, d751002, Shanghai, China).

### Immunohistochemistry and picric-sirius red (PSR) staining

Ninety rat liver samples were embedded into a tissue microarray by Outdo Biotech (Shanghai, China), stained with diaminobenzidine (Vector Laboratories, SK-4100), and counterstained with hematoxylin (Thermo Scientific, 7211, USA) to visualize the immunoreaction product following the manufacturers' suggested protocols. Antibodies used are listed in Supplementary Table 2. Liver sections were incubated with PSR solution for 1 h then hematoxylin for 5 min to dye the nuclei. Quantification of Ki67-positive hepatocytes and PSR area were performed by blinded manual counting of five random visual fields (200×).

### Immunofluorescence assay

Liver tissue sections or cells were permeabilized with 0.1% Triton X-100 and then incubated with TGFβ1, α smooth muscle actin (αSMA), hepatocyte nuclear factor α (HNF4α), and GFP primary antibodies. After washing with PBS, the samples were incubated with goat anti-mouse IgG and goat anti-rabbit IgG secondary antibodies. Antifade reagent with the nuclear stain Hoechst (Invitrogen, P36985) was added before imaging. Images were captured on a fluorescence microscope.

### Statistical analyses

All statistical analyses were blindly performed. Data are presented as mean ± standard deviation. Differences between groups were assessed by Mann-Whitney U Test. Shapiro-Wilk method was used to test if the data were Gaussian distributed. Two-tailed ANOVA was used for multiple testing and Šidák method was used to justify the data. Statistical analyses were performed using Prism v8.0 (GraphPad, San Diego, CA). *P* < 0.05 was considered statistically significant. Statistical significances are presented as *P < 0.05, **P < 0.01, and ***P < 0.001.

## Results

### ALPPS-associated liver regeneration was attenuated in fibrotic rats

To mimic the ALPPS process in fibrotic liver, CCl_4_ or DEN were used to induce liver fibrosis in two groups of rats. Histological samples were routinely stained for H&E, PSR, and immunohistochemistry were graded for degree of liver fibrosis by 2 different pathologists using the METAVIR scoring system: 0 (no fibrosis), 1 (mild fibrosis), 2 (moderate fibrosis), 3 (severe fibrosis), and 4 (cirrhosis) 15. The severity of liver fibrosis increased with treatment duration (Figure S1A). CCl_4_ was more efficient than DEN in inducing liver fibrosis with higher score deviation; however, the fibrosis score was consistently 2/3 on week 12 in the DEN-treated group (Figure S1A).

**Figure 1:**
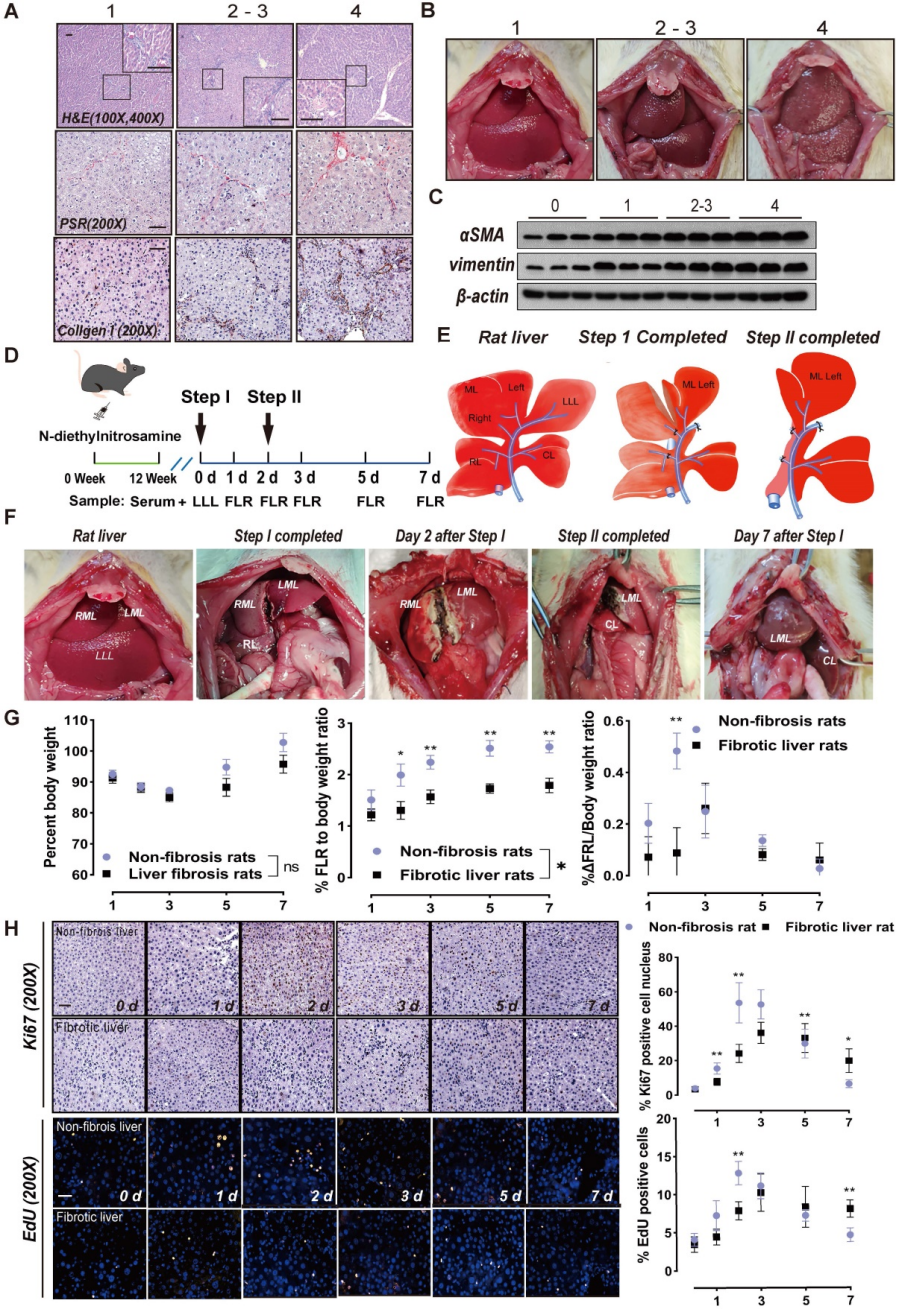
** ALPPS surgery in rats and its effects on liver regeneration.** (A) Representative images of paraffin-embedded liver tissue sections stained for H&E, PSR, and collagen I with METAVIR scores indicated (scale bars, 50 µm). (B) Representative images of rat livers with the indicated METAVIR scores. (C) Immunoblots of αSMA and vimentin in liver tissue. (D) Experiment design: Rats were treated with DEN for 12 weeks and then ALPPS was performed. The rats were sacrificed at the indicated timepoints following Step I before or after Step II (n = 4). (E) Illustrations of the anatomy of rat liver lobes (left) and procedures during Step I (middle) and Step II (right) of ALPPS. Step I included a hepatectomy of the LLL, ligation of the portal vein branches of the RL and RML, and partitioning of the liver parenchyma of the ML. The LML and CL were the FLR (26-30% of the total liver volume). A relaparotomy was performed in Step II 48 h later following a significant increase in FLR volume. A hepatectomy was performed to remove the deportalized liver lobes (RML and RL). (F) Representative photographs of FLR collected at the indicated timepoints after Step I before or after Step II. (G) BW (left), FLR/BW (middle), and ΔFLR/BW (right) of rats with non-fibrotic and fibrotic livers following ALPPS procedures (n = 4, two-tailed ANOVA). (H) Ki67 and EdU staining of paraffin-sectioned non-fibrotic and fibrotic liver tissues to assess liver regeneration (scale bars, 50 µm; n = 4, two-tailed ANOVA).

When fibrosis was severe, H&E staining demonstrated that the liver lobular structure was damaged and the hepatocytes were irregularly arranged. Further, the normal hepatic lobule structure almost disappeared, forming clear pseudo-lobules (Figure 1A). Histological analysis of collagen fibers using PSR and collagen I staining revealed that fibrosis was accompanied by an increase in fibrin (Figure 1A). When the fibrosis score was 2/3, there was no significant change in the surface of the liver, whereas multiple nodules could be seen with liver cirrhosis (Figure 1B). αSMA is a marker of HSC activation 21, while vimentin is mainly distributed in HSCs 22. With progression of liver fibrosis, the levels of these two proteins increased in liver tissues (Figure 1C).

Rats treated with DEN for 12 weeks (METAVIR scoring 2/3) were subjected to ALPPS. The LLL was excised in Step I as a control, and the hyperplastic FLR and blood samples were harvested on days 1, 2, 3, 5, and 7 after Step I (Figure 1D). The ALPPS surgery procedure is described in Figure 1E. A photograph of a fibrotic rat undergoing ALPPS surgery is shown in Figure 1F, and photographs of harvested FLR at indicated timepoints before or after Step II are shown in Figure S1B. The FLR were paler in color and more swollen in texture than the LLLs.

The liver growth rate of non-fibrotic rats peaked on day 2 after Step I, whereas the peak of liver proliferation in fibrotic rats lagged to day 3 and the overall regeneration level decreased (Figure 1G). Meanwhile, ALT and ALB data suggest that postoperative liver function damage was more serious in fibrotic rats than non-fibrotic rats, but was similar to normal rats and gradually recovered (Figure S1C). This suggests that the liver function of fibrotic rats needs more time to recover. Liver regeneration was assessed using Ki67 and EdU staining. An increase in positive nuclei indicated that the liver cells had enhanced mitotic activity and were proliferating (Figure 1H).

### ALPPS-associated liver regeneration was related to the TGFβ1 signaling pathway

**Figure 2:**
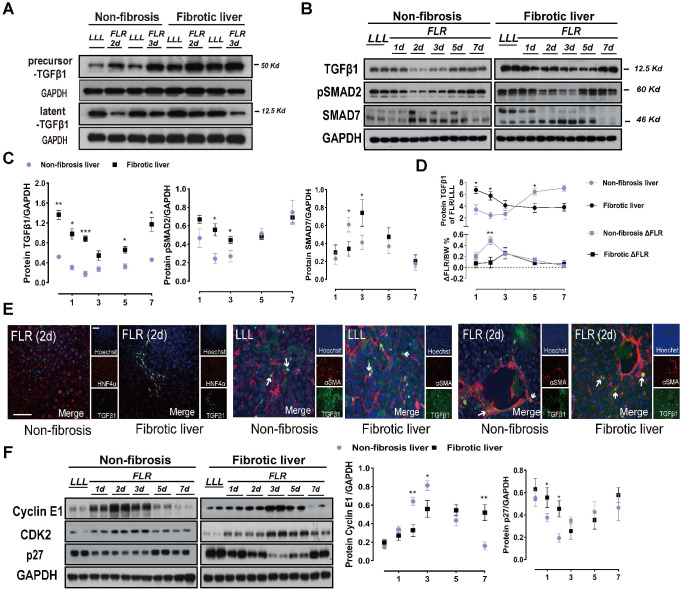
**TGFβ1 pathway components after ALPPS surgery.** (A) Immunoblots of precursor TGFβ1 and latent TGFβ1 proteins in liver tissues on days 2 and 3 after Step I. (B, C) Western blot analysis of TGFβ1 pathway components from lysates of non-fibrotic and fibrotic livers following ALPPS procedures at the indicated timepoints. GAPDH served as the loading control. The samples were derived from the same experiment and the blots were processed in parallel. (D) Statistical analysis of TGFβ1 levels and ΔFLR in fibrotic and non-fibrotic livers following ALPPS procedures at the indicated timepoints (n = 4, two-tailed ANOVA). Low peak expression of TGFβ1 protein corresponded to maximum ΔFLR in both groups. (E) TGFβ1/HNF4α and TGFβ1/αSMA immunofluorescence co-staining showing colocalization of TGFβ1 and αSMA in activated HSCs (400× magnification; scale bars, 50 µm). (F) Immunoblots of cyclin E1, CDK2, and p27 (cip) in liver tissues harvested at the indicated timepoints (n = 4, two-tailed ANOVA). GAPDH served as the loading control. The samples were derived from the same experiment and the blots were processed in parallel.

To describe the relevance of TGFβ1 in ALPPS of fibrotic liver, we measured the protein expressions of components related to the classical TGFβ1 pathway during ALPPS. TGFβ1 mRNA detected by qRT-PCR was upregulated after ALPPS in both the DEN-treated and control groups (Figure S2A). Interestingly, while increased precursor TGFβ1 was detected post-surgery by western blot, latent TGFβ1 (referred to as TGFβ1) declined to varying degrees in both groups at the indicated timepoints (Figure 2A-C).

Notably, the expression of TGFβ1 in liver tissues was closely related to the FLR growth kinetics (ΔFLR) (Figure 2D). For both DEN-treated and control groups, the expression of TGFβ1 was low at the peak ΔFLR. Phosphorylation of SMAD2, a main downstream effector molecule of TGFβ1, was downregulated with the change in TGFβ1 after ALPPS (Figure 2B, C). Dysregulation of other downstream protein components of the classical TGFβ pathway, such as SMD2, p-SMD3, SMAD4, and SMAD6, was not observed (Figure S2B). As a negative regulator of the TGFβ pathway, SMAD7 is believed to act on TGFβR1 and inhibit the phosphorylation of SMAD2 23. In our model, we found that the expression of SMAD7 was opposite that of pSMAD2, suggesting that SMAD7 may play a role in promoting liver regeneration in ALPPS (Figure 2B, C). Immunofluorescence TGFβ1/HNF4α co-staining of paraffin-embedded liver sections collected after surgery demonstrated that TGFβ1 was mainly located in non-parenchymal cells (Figure 2E). To assess the localization of TGFβ1 in activated HSCs, we performed TGFβ1/αSMA co-staining. TGFβ1 colocalized with αSMA (Figure 2E), indicating the presence of TGFβ1 in activated HSCs. Furthermore, TGFβ1 and αSMA colocalization remained stable over time (Figure 2E).

Unlike in humans, the rodent model of liver fibrosis gradually fades after drug withdrawal 24. To address this concern, we examined the expression levels of relevant proteins in a cohort of rats with DEN withdrawal without ALPPS to determine a baseline (Figure S2C).

TGFβ has been reported to block cell cycle in cancer and arrest tumor cells in the G1 phase 12. To verify the mechanism of TGFβ1 inhibition of hepatocyte regeneration, we detected G1/S-associated cyclin E1 and mRNA by qRT-PCR and western blot (Figure 2F, Figure S3A, B). Cyclin E1 controls the cell cycle transition from the G1 phase to the S phase by interacting with cyclin-dependent kinase 2 (CDK2). Together, they form a complex that triggers a signaling cascade that drives cells into the S phase. Western blots of cyclin E1 and CDK2 showed decreased protein levels in the fibrotic group after Step I (Figure 2F). Meanwhile, cyclin E1 and CDK2 were found to be suitable indicators of ALPPS-associated regeneration. The expression of p27 was opposite that of cyclin E1 and CDK2 (Figure 2F), indicating that p27 may play a negative role in ALPPS-associated liver regeneration. Hypothesizing that these changes in the expression levels of the TGFβ1 pathway may be caused by the specificity of DEN, we detected TGFβ1-related proteins in CCL4-treated rats after ALPPS and found similar changes (Figure S3C).

### AAV-shTGFβ1 accelerated regeneration of fibrotic livers during ALPPS

**Figure 3:**
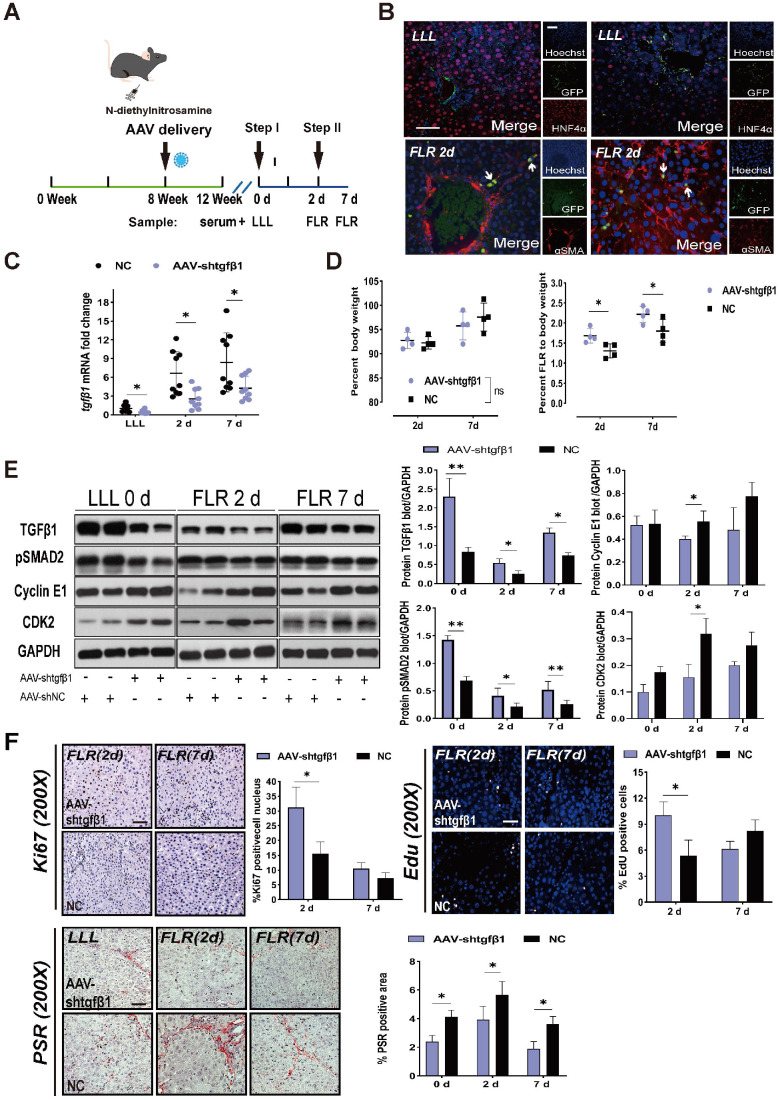
** AAV-shTGFβ1 accelerated fibrotic liver regeneration and improved fibrosis.** (A) Experiment design: DEN-treated rats were transfected with HSC-specific AAV-GFAP-shTGFβ1 on week 8. The rats were continuously treated with DEN until week 12 and then ALPPS was performed. The rats were sacrificed on days 2 and 7 after Step I (n = 4). NC rats were treated with AAV-shNC for 4 weeks. (B) Immunofluorescence GFP/αSMA and GFP/HNF4α co-staining confirming the specific transduction of AAV into activated HSCs before and after surgery (400× magnification; scale bars, 50 µm). (C) qRT-PCR analysis of TGFβ1 mRNA levels in liver tissue after knockdown with AAV-shTGFβ1 (n = 4, two-tailed ANOVA). (D) BW and FLR/BW at the indicated timepoints for AAV-shTGFβ1 and NC groups (n = 4, two-tailed ANOVA). (E) Hepatic protein expression (normalized to GAPDH) of TGFβ1, pSMAD2, cyclin E1, and CDK2 at the indicated timepoints (n = 4, two-tailed ANOVA). (F) Ki67, EdU, and PSR staining demonstrating accelerated liver regeneration and reduced fibrosis in the AAV-treated group (scale bars, 50 µm). The percentage of Ki67+ and EdU+ cells and the PSR-stained area were analyzed separately (n = 4, two-tailed ANOVA).

To further verify the relationship between TGFβ1 and ALPPS-related liver regeneration, we designed an HSC-specific AAV-GFAP-shTGFβ1 and transfected it into DEN-treated rats on week 8 through the portal vein. We continued to treat the rats with DEN until week 12 and then performed ALPPS (Figure 3A). Considering that the ratio of TGFβ1 mRNA was highest on day 7 (Figure S4) and the biggest difference in ΔFLR appeared on day 2 after surgery in fibrotic vs. non-fibrotic liver, we harvested liver samples on days 2 and 7 after Step I (n = 4) (Figure 3A). To demonstrate the specific transfection of AAV into HSCs, we used an immunofluorescence assay to measure GFP/αSMA and GFP/HNF4α colocalization, indicating the presence of GFP in activated HSCs (Figure 3B). The colocalization assay results in fibrotic LLL and FLR suggested that transfected GFP was present before and after the surgery (Figure 3B).

TGFβ1 mRNA knockdown was detected by qRT-PCR in LLL and FLR tissues (Figure 3C). Compared to the normal control (NC) group, rats treated with AAV-shTGFβ1 had significantly increased FLR regeneration (Figure 3D), decreased expression of pSMAD2, and increased expression of cyclin E1 and CDK2 (Figure 3E). The regenerative activity of the liver was found to be enhanced by EdU and Ki67 staining (Figure 3F). Collagen deposits in the liver tissues were visualized and quantified using PSR staining, which detects collagen types I and III. The collagen deposit area from DEN injury was significantly reduced by transfection with AAV-shTGFβ1 (Figure 3F). Admittedly, it is difficult to distinguish whether AAV-shTGFβ1 increased liver regeneration by inhibiting hepatic fibrosis or inhibiting TGFβ1.

### LY2157299 partially restored liver regeneration

**Figure 4:**
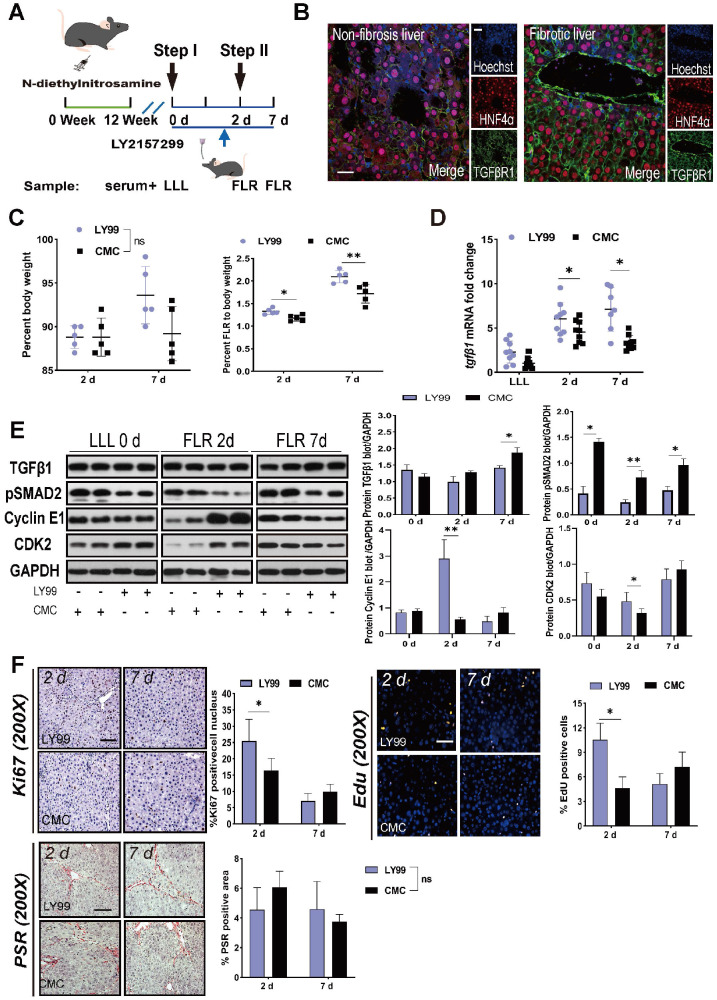
** Effects of LY2157299 on liver regeneration.** (A) Experiment design: Rats were treated with DEN for 12 weeks and then orally administered 150 mg/kg LY2157299 twice per day for 7 days before sacrifice. The rats were sacrificed on days 2 and 7 after Step I (n = 5). NC rats were administered 1% CMC intragastrically. (B) HNF4α/TGFβR1 co-staining of day 2 FLR tissue sections demonstrating that TGFβR1 was mainly located on the membrane of hepatocytes (scale bars, 50 µm). (C) BW and FLR/BW of rats treated with LY2157299 or CMC after surgery (n = 5, two-tailed ANOVA). (D) qRT-PCR analysis of TGFβ1 mRNA demonstrating upregulation in the LY2157299-treated group on days 2 and 7 post-surgery (n = 5, two-tailed ANOVA). (E) Hepatic protein expression (normalized to GAPDH) of TGFβ1, pSMAD2, cyclin E1, and CKD2 in the LY2157299 and CMC groups. (F) Representative images of liver tissue stained with Ki67, EdU, and PSR (scale bars, 50 µm). The percentage of Ki67+ and EdU+ cells and the PSR-stained area were analyzed separately (n = 4, two-tailed ANOVA). No significant difference in PSR-stained area was observed between the two groups.

To elucidate the targets of TGFβ1 in the inhibition of ALPPS-associated regeneration, we applied LY2157299 to specifically inhibit TGFβR1 *in vivo*. After treatment with DEN for 12 weeks, rats were given LY2157299 (150 mg/kg) or CMC (control) orally twice per day for 7 days before sacrifice (Figure 4A). The rats were sacrificed on days 2 and 7 after Step I (n = 5) (Figure 4A). Immunofluorescence HNF4α/TGFβR1 co-staining of FLR tissues (day 2) (Figure 4B) and hepatocytes (Figure S5) demonstrated that TGFβR1 was mainly located on the membrane of hepatocytes. Compared to the CMC group, rats treated with LY2157299 had significantly increased FLR regeneration (Figure 4C). TGFβ1 mRNA detected by qRT-PCR was upregulated on days 2 and 7 (Figure 4D). Compared to the control group, rats treated with LY2157299 had unchanged protein expression of TGFβ1 on day 2 (increased on day 7); however, expression of the downstream protein pSMAD2 was significantly decreased (Figure 4E), indicating that the TGFβ1/pSMAD2 pathway was inhibited and TGFβ1 mRNA might be upregulated in compensation (Figure 4D). Cyclin E1 and CDK2 levels were also increased in the LY2157299 treated animals (Figure 4E). The regenerative activity of the fibrotic liver was found to be enhanced by EdU and Ki67 staining (Figure 4F). PSR staining performed on paraffin sections of liver tissues showed no significant difference between the two groups (Figure 4F). In the LY2157299-treated rats, proliferation of the fibrotic liver was accelerated without reduction of fibrosis, suggesting that reduction of fibrosis and inhibition of TGFβ1 both play positive roles in liver regeneration.

### Coadministration of AAV-shTGFβ1 and LY2157299 achieved stronger regeneration than monotherapy

**Figure 5:**
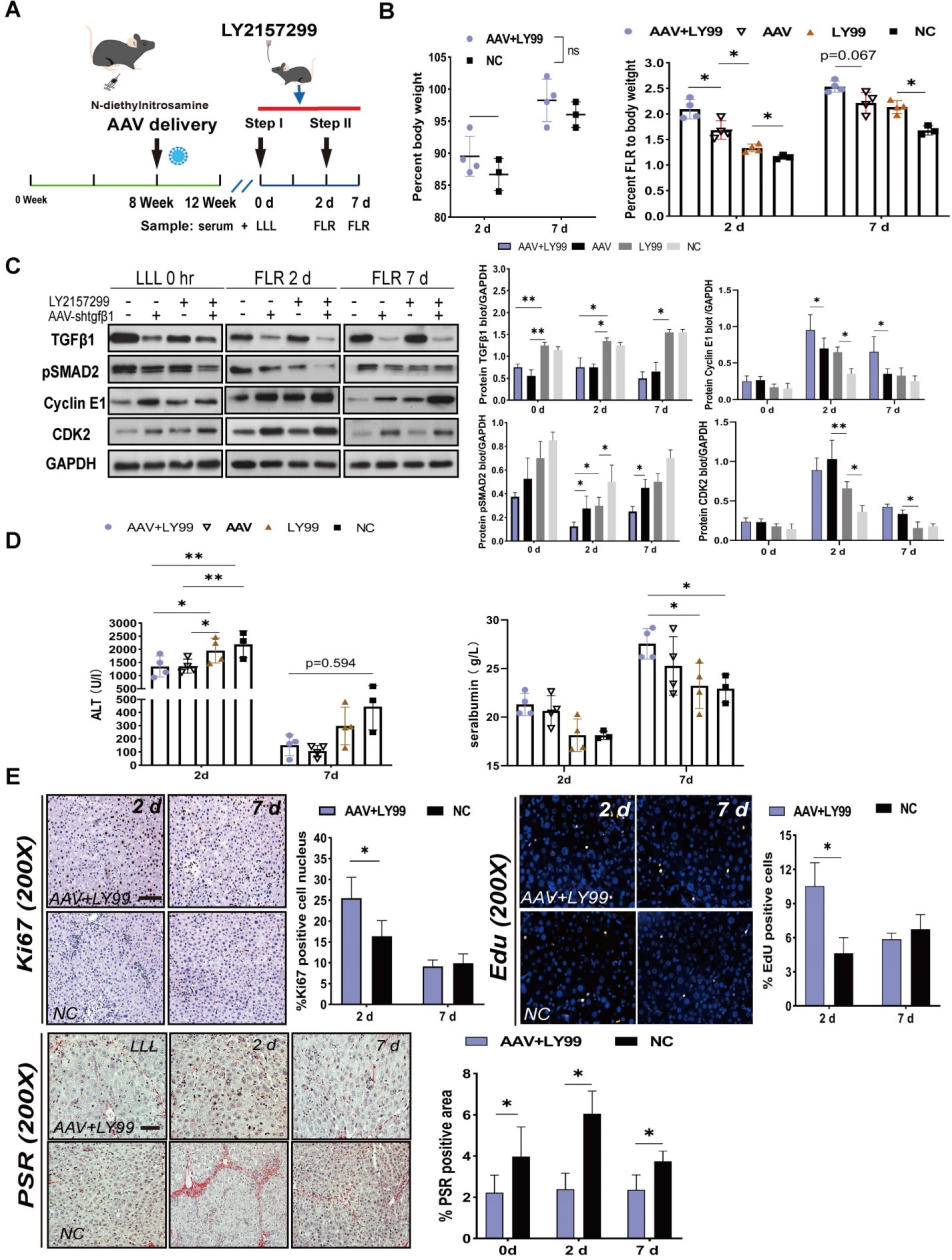
** Effects of AAV-shTGFβ1 and LY2157299 combination treatment on liver regeneration.** (A) Experiment design: Rats were treated with DEN and then transfected with AAV on week 8. The rats were continuously treated with DEN until week 12. LY2157299 was administered 7 days before sacrifice. The rats were sacrificed on days 2 and 7 after Step I (n = 4). NC rats were treated with AAV-shNC and CMC (n = 3). (B) BW and FLR/BW after surgery (n = 3-5, two-tailed ANOVA). (C) Hepatic protein expression (normalized to GAPDH) of TGFβ1, pSMAD2, cyclin E1, and CKD2 in liver tissue from the combination therapy, monotherapy, and NC groups (n = 3-5, two-tailed ANOVA). (D) Serum ALT and ALB levels on days 2 and 7 after surgery. (E) Ki67, EdU, and PSR staining demonstrating accelerated liver regeneration and reduced fibrosis in the combination therapy group (Scale bars, 50 µm; n = 4, two-tailed ANOVA).

Since AAV-shTGFβ1 and inhibited the TGFβ1/pSMAD2 pathway by downregulating TGFβ1 mRNA and suppressing TGFβR1, we hypothesized that coadministration of AAV-shTGFβ1 and LY2157299 would produce additive effect. DEN-treated rats were transfected with AAV on week 8 and continuedly treated with DEN until week 12. LY2157299 was administered 7 days before sacrifice (Figure 5A). The doses of virus and drug were the same as those used for monotherapy. Rats cotreated with AAV and LY2157299 had stronger liver regeneration on day 2 after surgery (2.09 ± 0.19) compared to rats treated with AAV monotherapy (1.68 ± 0.18), LY2157299 monotherapy (1.33 ± 0.07), or NC (1.15 ± 0.06) (*P* < 0.05 for combination therapy vs. AAV, *P* < 0.05 for AAV vs. LY2157299; n = 4) (Figure 5B). Rats cotreated with AAV and LY2157299 or AAV monotherapy had the strongest liver regeneration on day 7 after surgery (2.54 ± 0.11 and 2.21 ± 0.20) compared to rats treated with LY2157299 monotherapy (2.13 ± 0.12) or NC (1.68 ± 0.09) (*P* < 0.05 for AAV vs. LY2157299; n = 3-5; two-way ANOVA) (Figure 5B). This result is consistent with the protein expressions of TGFβ1, pSMAD2, cyclin E1, and CDK2 detected by western blot (Figure 5C).

To assess liver function after ALPPS, we measured ALT and ALB serum levels at the indicated timepoints (Figure 5D). Interestingly, the ALT data on day 2 showed that the liver function of the AAV-treated group was better than that of the LY2157299-treated group, the latter being not significantly different from the NC group (Figure 5D), suggesting that weakening of liver fibrosis, not inhibition of TGFβ1, improves liver function. The regenerative activity of the liver was assessed by EdU and Ki67 staining, and liver fibrosis was assessed by the percentage of PSR-positive area (Figure 5E). In summary, our data suggests that the combined treatment of AAV-shTGFβ1 and LY2157299 elicited a stronger regenerative effect than monotherapy. This may be because TGFβR1 inhibition activated upstream TGFβ1 expression. Therefore, the combination of both drug and virus is likely to be particularly potent.

### Inhibition of TGFβ1/pSMAD2 accelerated hepatocyte proliferation *in vitro*

**Figure 6:**
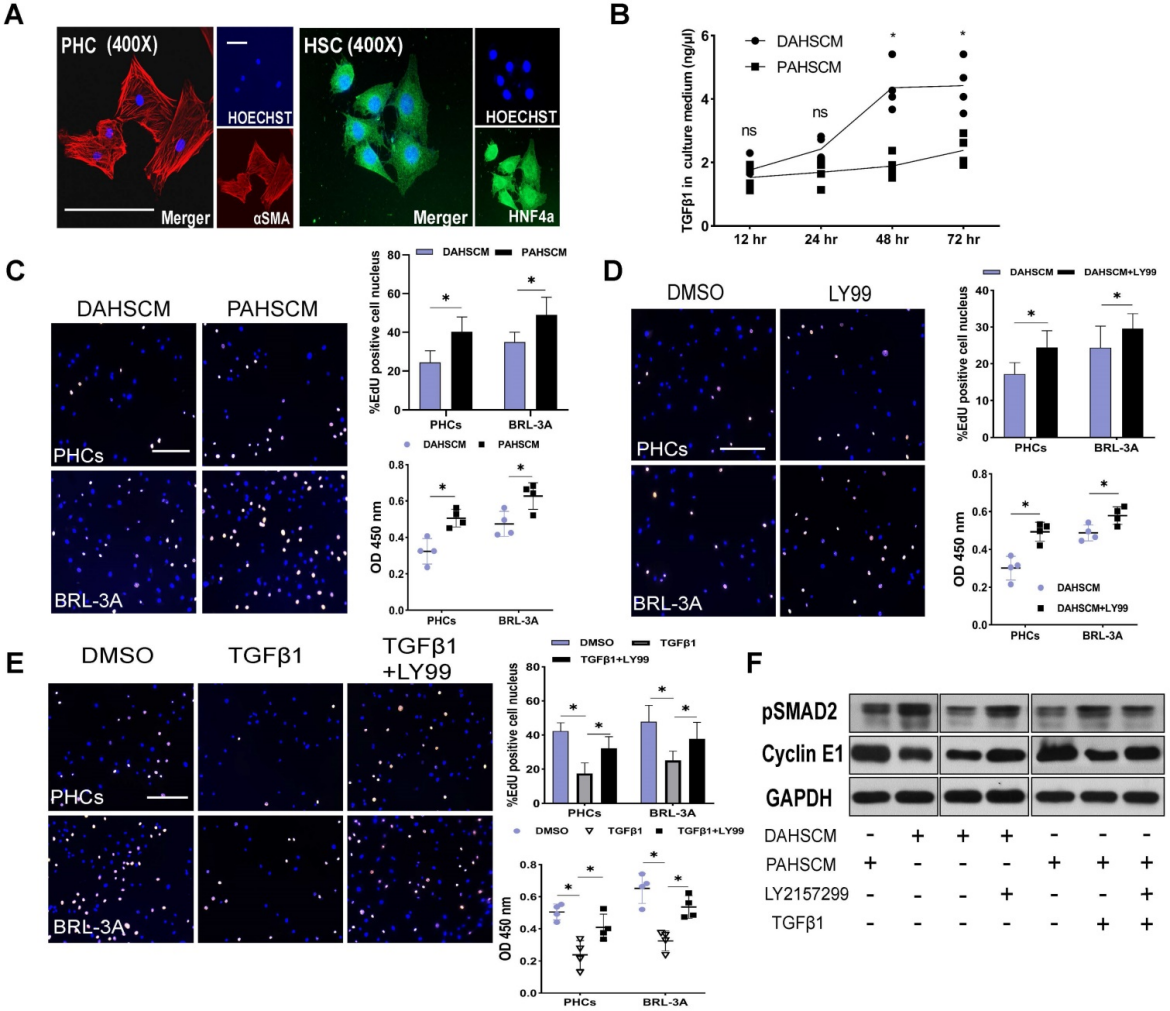
** TGFβ1 affects hepatocyte proliferation *in vitro*.** (A) HNF4α and αSMA staining of PHCs isolated from DEN-treated rats and primary HSCs isolated from DEN- and PBS-treated rats on day 2 after ALPPS following 24 h culture (scale bars, 10 µm). (B) ELISA assay of TGFβ1 concentration in DAHSCM and PAHSCM at the indicated timepoints (n = 4, two-tailed ANOVA). (C) EdU staining and CCK8 assay of HPCs and BRL-3A cells after 24 h co-culture in DAHSCM or PAHSCM to assess proliferation. (D) Proliferation of co-cultured cells in DAHSCM with LY2157299 or DMSO. (E) Proliferation of co-cultured cells in PAHSCM with TGFβ1, TGFβ1 and LY2157299, or DMSO (scale bars, 100 µm; n = 4, Mann-Whitney U Test). (F) Western blots of pSMAD2 and cyclin E1 in HPCs cultured in PAHSCM, DAHSCM, DAHSCM with LY2157299, PAHSCM with TGFβ1, or PAHSCM with TGFβ1 and LY2157299 for 24 h. GAPDH served as the loading control.

To elucidate the relationship between HSC-derived TGFβ1 and hepatocyte proliferation, we isolated HSCs and PHCs from DEN-treated and PBS-treated rats 48 h after ALPPS surgery using previously reported methods 19, 20. After 24 h of culture, adherent cells were identified by HNF4α and αSMA fluorescence staining (Figure 6A). An ELISA assay was used to measure the concentration of protein TGFβ1 in the culture medium of DEN-ALPPS treated rat primary HSCs (DAPHSCM) and PBS-ALPPS treated rat primary hepatic stellate cells culture medium (PAPHSCM) at the indicated timepoints. There was a significant difference between the two groups in the concentration of active TGFβ1 at 48 and 72 h (48 h: 4.36 ± 0.75 in DAPHSCM 1.89 ± 0.36 in PAPHSCM, n = 4, *P* = 0.01; 72 h: 4.42 ± 0.80 in DAPHSCM, 2.38 ± 0.47 in PAPHSCM, *P* = 0.03, n = 4) (Figure 6B). This data suggests that 48 h culture medium can be used for subsequent co-cultivation.

After 24 incubation with DAPHSCM or PAPHSCM, HPCs and BRL-3A cells showed a significant change in proliferation (Figure 6C). The proliferation inhibition in DAHSCM was partially alleviated by 10 μM LY217299 (Figure 6D). CCK-8 and EdU assays demonstrated that rTGFβ1 added to PAHSCM significantly inhibited the proliferation of HPCs and BRL-3A cells, whereas LY2157299 partially alleviated the inhibition (Figure 6E). Moreover, detection of pSMAD2/cyclin E1 protein in HPCs by western blot in gain- or loss-of function experiments (Figure 6F) confirmed that the TGFβ1/pSMAD2 pathway inhibited hepatocyte proliferation.

## Discussion

The ALPPS procedure, which was discovered by chance in 2007, showed that the intrinsic capacity of the liver to regenerate is far greater than traditionally believed 4. Compared to conventional two-staged hepatectomy, greater hypertrophy can be achieved with ALPPS. Therefore, it is a novel treatment strategy for patients with primary unresectable liver tumors. Further, because ALPPS can be utilized *in situ*ations with predicted small volumes of FLR, it is a treatment option for patients with liver fibrosis or cirrhosis. For example, one study showed that regeneration of cirrhotic livers after ALPPS, though attenuated compared to non-fibrotic livers, was still significantly higher than after standard PVL 25. Likewise, another study found that ALPPS can be a viable treatment option for patients with hepatitis B virus-related hepatocellular carcinoma. Patient FLR volumes after ALPPS increased by an average of 56.8% before Step II, and 91.1% of patients underwent Step II surgery after an average of 12 days (6-28 days) (9). However, while increased growth of the FLR is the major benefit of the ALPPS procedure, it has been described as a double-edged sword 26-28. Enthusiasm has been tempered by high morbidity and mortality rates. Therefore, care must be taken in patient selection. Consequently, understanding the mechanisms responsible for the success of ALPPS is paramount for proper patient selection.

ALPPS-induced regeneration is believed to be the result of altered liver hemodynamics induced by the ligation of the hepatic portal vein, inflammation caused by the liver partition, and fluctuations in the expression of regeneration-related signaling pathways. Regarding the latter, our group's previous work found that there was an increase in the expression levels of interleukin-6 (IL-6), tumor necrosis factor α (TNF-α), and YAP in the FLR tissue of rats following ALPPS 29. IL-6 and TNF-α play important roles in the initial stages of liver regeneration. These two pro-inflammatory cytokines are produced by activated Kupffer cells in the liver and promote the transition of liver cells from the G0 to the G1 phase of the cell cycle 30. Expression of the cyclin E/CDK2 complex also increased in the FLR tissue, but the upstream mechanism remains unclear 29. Innovatively, Schlegel et al. mimicked human ALPPS procedures in mice, which laid the foundation for animal research on ALPPS 31. Their further studies showed that early JNK1 activity can induce IHH release from HSCs, which promotes the GLI1-CCND1 axis in hepatocytes to accelerate liver regeneration 32, 33. Because these studies were performed in healthy liver tissue, in this study we examined the molecular changes associated with ALPPS in fibrotic livers.

Liver fibrosis is mediated by the TGFβ signaling pathway, which also mediates lung and kidney fibrosis 13, 34. We hypothesized that it is activation of the TGFβ pathway that leads to attenuation of ALPPS-associated hepatocyte regeneration.

To test this hypothesis, we generated a rat model of liver fibrosis by treating rats with DEN for 12 weeks (METAVIR system scores 2/3). According to estimates of the formation of liver fibrosis and cancer, DEN seems to produce a suitable animal model for analyzing certain aspects and processes that can promote the pathogenesis of human hepatocellular carcinoma 35. We established a reproducible DEN animal model for further research on oncological outcomes in the DEN-ALPPS-TGFβ1(-) rats.

When activated, TGFβ1 binds to its receptor, which in turn results in phosphorylation of SMAD2. This results in a complex signaling cascade that prevents cyclin E1 binding to CDK2, which subsequently prevents hepatocytes from exiting the G1 phase and transitioning into the S phase. In our study, knocking down TGFβ1 expression by transfection with AAV-shTGFβ1 or inhibiting TGFβR1 activity using LY2157299 reduced pSMAD2 expression *in vivo*. This resulted in increased cyclin E1 and CDK2 and enhanced liver regeneration. Astonishingly, these interventions accelerated ALPPS-associated liver regeneration and improved liver function in all rats with varying degrees of hepatic fibrosis. These results are concordant with previously published reports. Nakamura et al. found that TGFβ1 inhibition in rats subjected to dimethylnitrosamine resulted in reduced hepatocyte apoptosis, upregulation of multiple growth factors, and liver regeneration 36. Hu et al. found that simultaneous deletion of cyclin E1 and CDK2 in mice resulted in reduced *in vivo* hepatocyte proliferation and liver regeneration after partial hepatectomy 37. In hepatocyte-specific TGFβR2 knockout mice with 70% hepatectomy, inhibition of TGFβ1/TGFβR2 promoted hepatocyte proliferation, increased the volume of the liver after surgery, and increased the expression of cyclin E and SMAD2 38. The expression of SMAD2 was not increased in our study, which may be due to the suppression of TGFβR1 and blocking of SMAD2 phosphorylation in our model. Hepatocytes isolated from liver-specific TGFβR2 knockout mice with 70% hepatectomy were refractory to the growth inhibitory effects of TGFβ1 39. Hepatocyte proliferation returned to baseline 5 days after partial hepatectomy. This conclusion is consistent with our data. Although the TGFβ1 pathway was inhibited, the acceleration of regeneration stopped when the liver regenerated close to normal volume. This suggests that TGFβ1 is not necessary for the termination of regeneration. It was reported that TGFβ inhibition restores regenerative response by suppressing senescence 40. To observe the relationship between TGFβ and cell senescence in our model, we performed immunohistochemical staining of p21 and pSMAD2 in paraffin sections of livers 2 days after ALPPS (Figure S6A). We found that p21 and pSMAD2 were highly expressed in areas away from the central lobular vein, and fibrosis aggravated their expression. Detection of p21 protein by western blot (Figure S6B) showed that p21 was downregulated during liver regeneration, suggesting that inhibiting TGFβ1 may suppress cell senescence and accelerate liver regeneration.

Nonetheless, it appears some TGFβ1 signaling is necessary for liver regeneration. Oh et al. showed that knockout of Hippo-Yap, a signaling pathway that crosstalks with TGFβ and prevents hepatocytes from undergoing an EMT-like response, is necessary for liver regeneration 41. Interestingly, we found that the levels of TGFβ1 mRNA and precursor TGFβ1 increased whereas the expression of latent TGFβ1 protein decreased after ALPPS. This contradicts previous results that found an increase in TGFβ expression post-hepatectomy 42, 43. Possible explanations for this discrepancy are that ALPPS may interfere with the processing of latent TGFβ1 or latent TGFβ1 may exit the liver and enter the circulation.

We chose to use AAV-shTGFβ1 and LY2157299 to antagonize TGFβ signaling for three reasons: First, they have different targets (TGFβ1 mRNA and TGFβR1). We used this difference to verify if TGFβR1 is responsible for liver regeneration during ALPPS. Second, the transfection efficiency of AAVs was reported to be unaffected by cirrhotic liver 44. Third, our study showed that the expression level of pSMAD2 plays a key role in liver regeneration via TGFβ1 downstream signaling following ALPPS (Figure 3), and LY2157299 specifically inhibits phosphorylation of SMAD2 by inhibiting TGFβR1 23. By targeting the TGFβ1 pathway at separate steps in the signaling cascade and at different time points during the ALPPS procedures, we were able to increase our confidence that TGFβ1 signaling is vital for the success or failure of ALPPS. Our results strongly suggest this because both treatments improved regeneration of the FLR. However, only the AAV treatment inhibited the progression of liver fibrosis. LY2157299 had no significant influence on total liver fibrosis. A possible explanation for these findings is that liver fibrosis is a chronic and reversible process. Therefore, 4 weeks of AAV treatment may be required for tissue remodeling. Another possible explanation is that because LY2157299 inhibits TGFβR1, TGFβ1 may still promote fibrosis through non-pSMAD2-mediated pathways. Studies have shown that TGFβ1 crosstalks with BMP7, BMP9, and SMAD1/5/8, all of which can affect liver fibrosis 45-47.

Nevertheless, some care needs to be taken when interpreting our results. Animal research on ALPPS mainly focuses on regeneration and oncological effects. While accelerating liver regeneration, inhibiting tumor progression or at least not promoting tumor progression is a reasonable intervention for ALPPS. We need to further explore the effect of TGFβ1 pathway inhibition on liver tumor. Notably, ALPPS is associated with significant mortality. Because of this, we developed a reliable ALPPS model in rats with fibrotic livers. By retaining complete blood supply to the FLR lobes CL and LML (26-30% of the total liver volume), as well as the integrity of the hepatic artery and LML hepatic vein when ligating the portal vein branches and partitioning the liver parenchyma in Step I, we were able to ensure that the survival of the animals was above 90%.

In conclusion, we have established a rat ALPPS model of DEN-induced liver fibrosis with high survival and reproducibility. More importantly, we discovered that the TGFβ1/pSMAD2 pathway is involved in hepatocyte regeneration following ALPPS. Therefore, therapies designed to modulate TGFβ1/pSMAD2 constitute a promising adjuvant therapy for ALPPS.

## Supplementary Material

Supplementary figures, tables, and methods.

## Abbreviations

αSMA: α smooth muscle actin
AAV: adeno-associated virus
ALPPS: associating liver partition and portal vein ligation for staged hepatectomy
ALB: albumin
ALT: alanine aminotransferase
BW: body weight
CDK2: cyclin-dependent kinase 2
CL: caudate lobe
CMC: sodium carboxymethyl cellulose
DEN: *N*-diethylnitrosamine
DM: DEN- and ALPPS-treated rat primary HSCs culture medium
FBS: fetal bovine serum
FLR: functional liver remnant
H&E: hematoxylin and eosin
HNF4α: hepatocyte nuclear factor α
HSCs: hepatic stellate cells
LLL: left lateral lobe
LML: left middle lobe
ML: middle lobe
NC: negative control
PBS: phosphate-buffered saline
PHCs: primary hepatocytes
PAHSCM: PBS- and ALPPS-treated rat primary HSCs culture medium
PSR: picric-sirius red
PVE: portal vein embolization
PVL: portal vein ligation
RL: right lobe
RML: right middle lobes
SMAD2: mothers against decapentaplegic homolog 2
TBIL: total bilirubin
TGFβ1: transforming growth factor beta 1
TGFβR1: transforming growth factor beta receptor 1
