# Supplementary material for: Inhibition of TGFβ1 accelerates regeneration of fibrotic rat liver elicited by a novel two-staged hepatectomy
Source: Theranostics. 2021 Mar 4;11(10):4743–58. doi: 10.7150/thno.52102 (PMC7978300; doi:10.7150/thno.52102)
Supplement: Supplementary file 1 — Supplementary figures, tables, and methods. [file thnov11p4743s1.pdf]

Figure S1

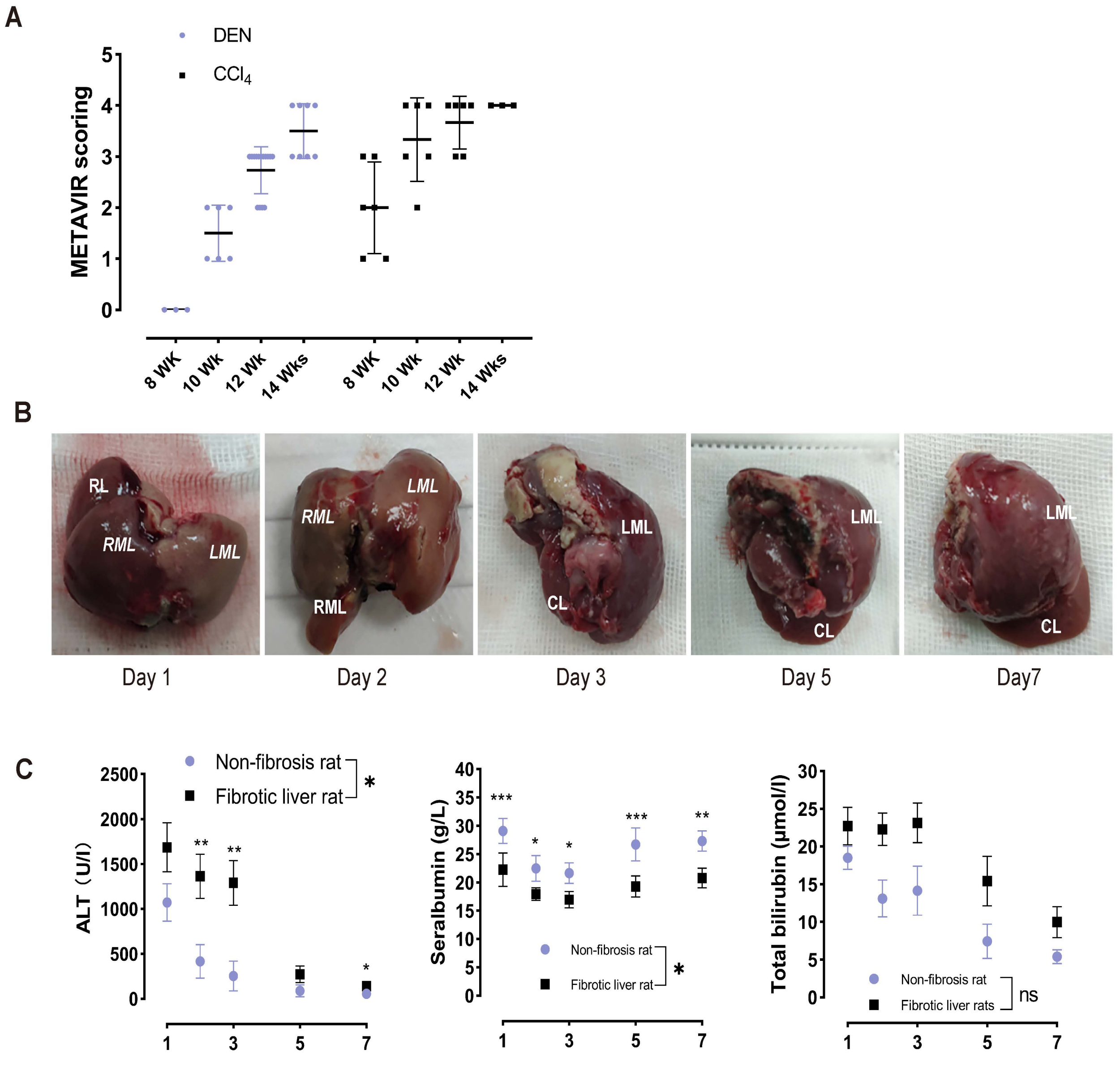
A
DEN
CCl_4
METAVIR scoring
5
4
3
2
1
0
8 WK
10 Wk
12 Wk
14 Wks
8 WK
10 Wk
12 Wk
14 Wks
B
RL
RML
LML
RML
LML
RML
LML
CL
LML
CL
LML
CL
Day 1
Day 2
Day 3
Day 5
Day7
C
Non-fibrosis rat
Fibrotic liver rat
*
ALT（U/l）
2500
2000
1500
1000
500
0
**
**
*
1
3
5
7
Seralbumin (g/L)
40
35
30
25
20
15
10
5
0
***
*
*
***
**
Non-fibrosis rat
Fibrotic liver rat
*
Total bilirubin (μmol/l)
30
25
20
15
10
5
0
Non-fibrosis rat
Fibrotic liver rats
ns

Figure S2

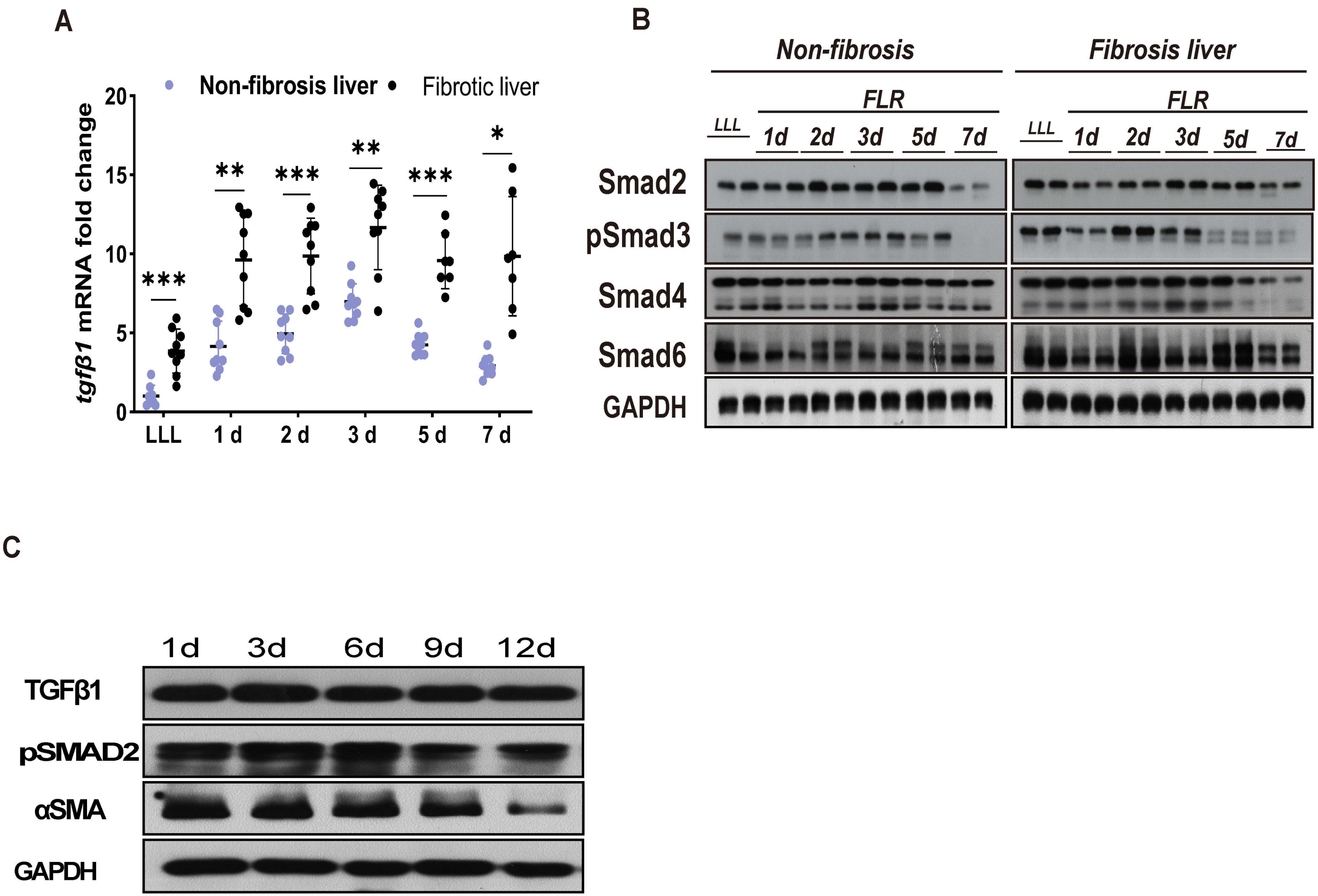
A
Non-fibrosis liver
Fibrotic liver
tgfβ1 mRNA fold change
20
15
10
5
0
***
**
***
**
***
*
LLL
1 d
2 d
3 d
5 d
7 d
B
Non-fibrosis
Fibrosis liver
FLR
FLR
LLL
1d
2d
3d
5d
7d
LLL
1d
2d
3d
5d
7d
Smad2
pSmad3
Smad4
Smad6
GAPDH
C
1d
3d
6d
9d
12d
TGFβ1
pSMAD2
αSMA
GAPDH

Figure S3

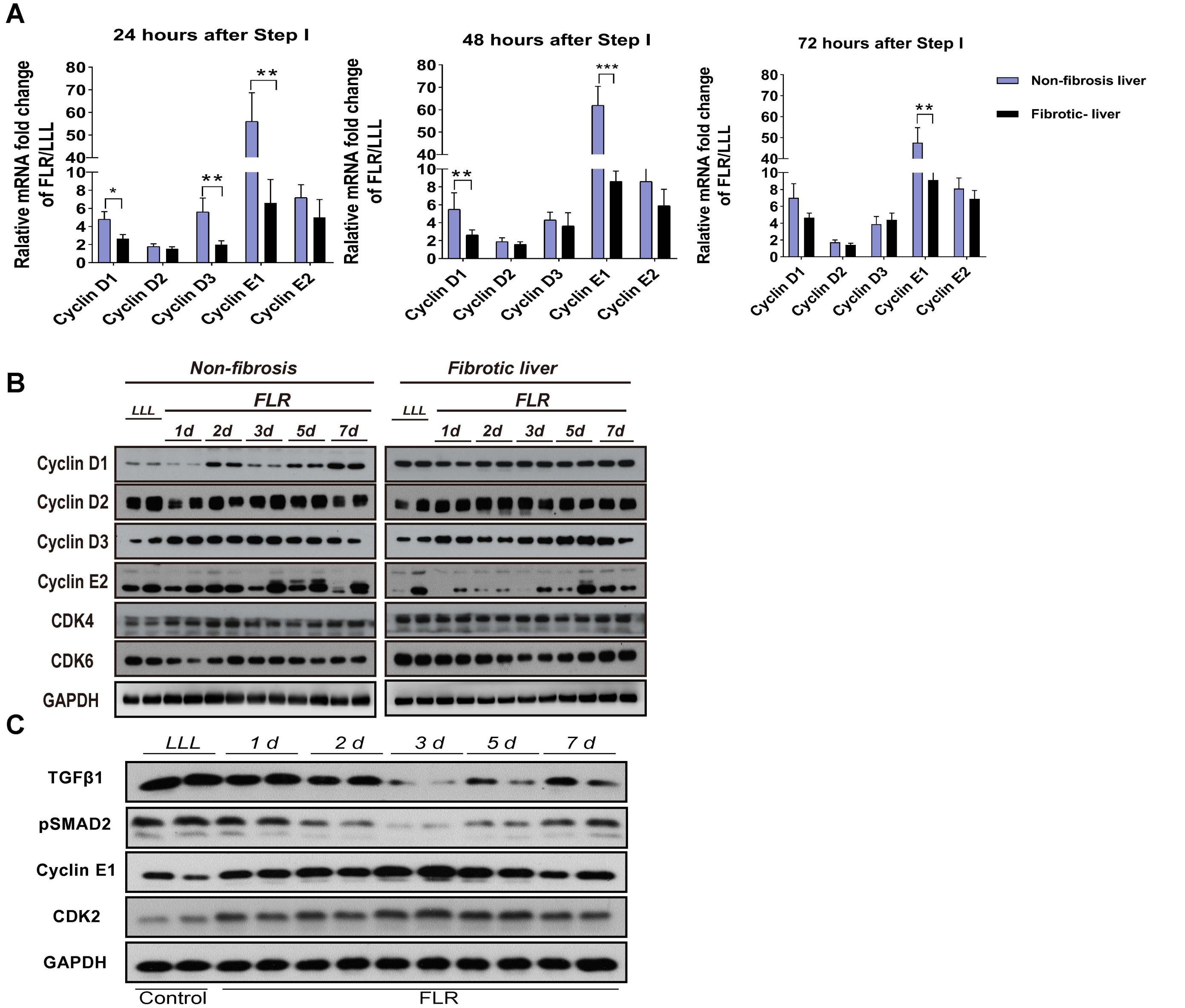
A
24 hours after Step I
48 hours after Step I
72 hours after Step I
Ralative mRNA fold change of FLR/LLL
Non-fibrosis liver
Fibrotic- liver
Cyclin D1
Cyclin D2
Cyclin D3
Cyclin E1
Cyclin E2
B
Non-fibrosis
Fibrotic liver
LLL
FLR
1d
2d
3d
5d
7d
Cyclin D1
Cyclin D2
Cyclin D3
Cyclin E2
CDK4
CDK6
GAPDH
C
LLL
1 d
2 d
3 d
5 d
7 d
TGFβ1
pSMAD2
Cyclin E1
CDK2
GAPDH
Control
FLR

Figure S4

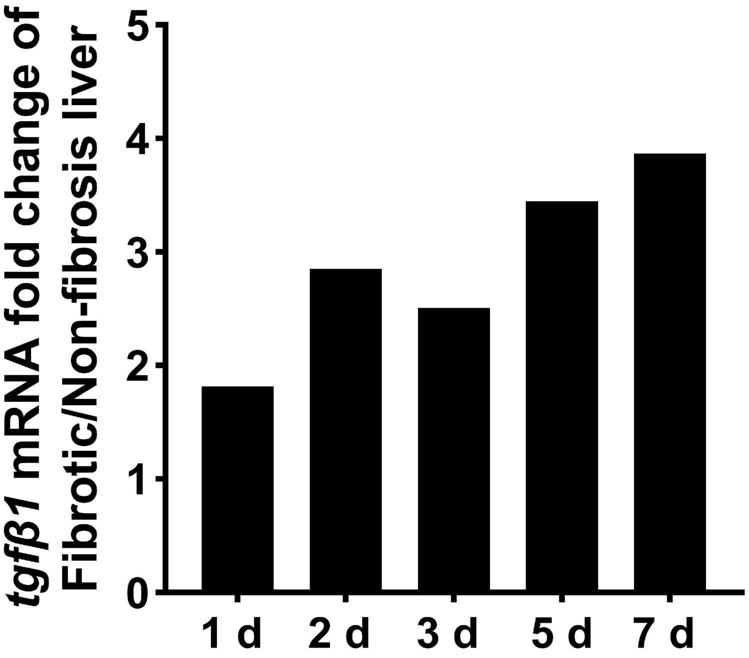
tgfβ1 mRNA fold change of
Fibrotic/Non-fibrosis liver
5
4
3
2
1
0
1 d
2 d
3 d
5 d
7 d

Figure S5

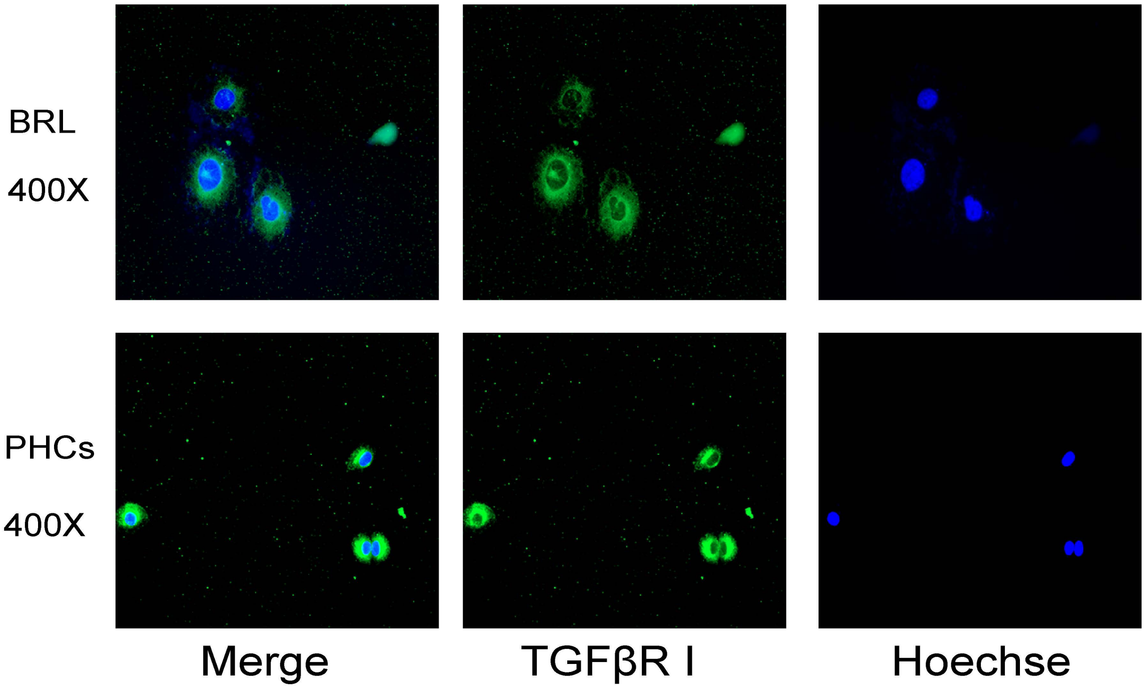

Figure S6

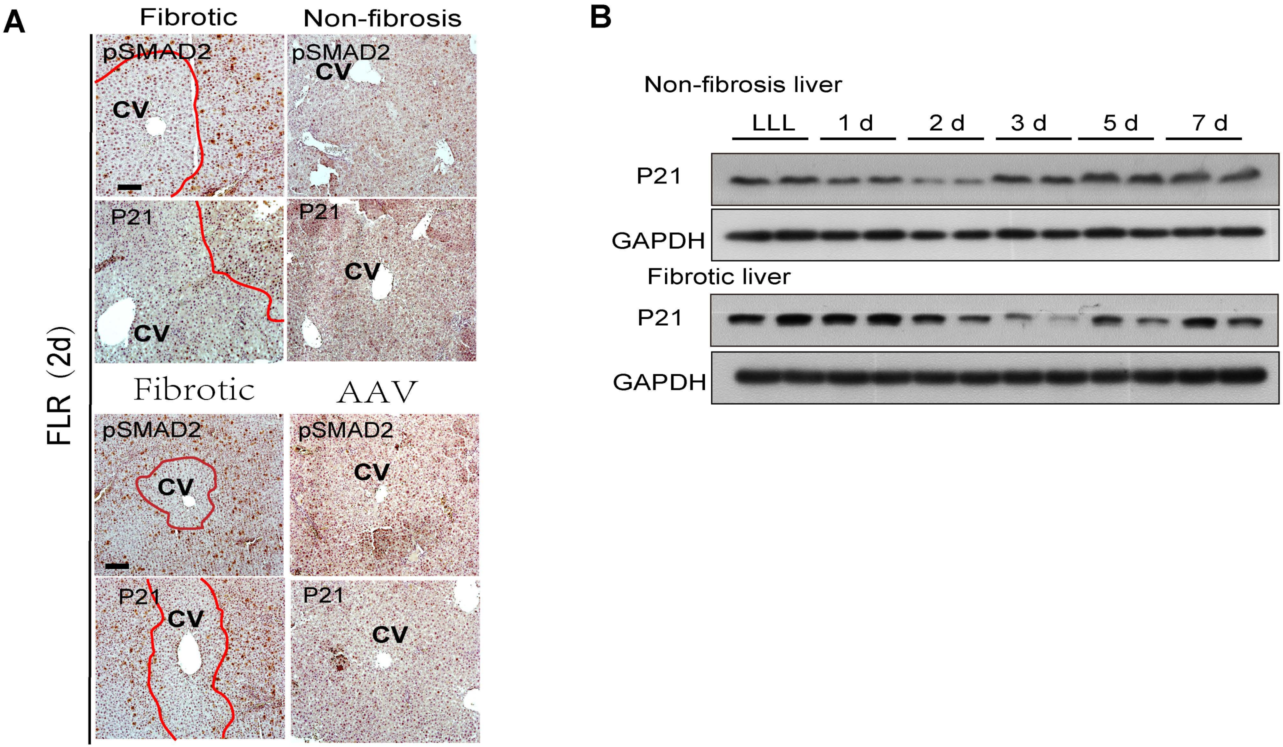
A
Fibrotic
Non-fibrosis
pSMAD2
CV
P21
FLR (2d)
Fibrotic
AAV
B
Non-fibrosis liver
LLL
1 d
2 d
3 d
5 d
7 d
P21
GAPDH
Fibrotic liver

Fig S1. ALPPS was performed on fibrotic livers rat. (A) Metavir scores of N-Nitrosodiethylamine-induced liver fibrosis and CCl_4-induced liver fibrosis at indicated time (n = 3-20). (B) Representative images of DEN treated livers at indicated time after ALPPS with or without Step II. (C) Serum ALT (left), ALB (middle), and TBIL (right) levels at the indicated timepoints (n = 4, two-tailed ANOVA).

Fig S2. TGFβ1 and -CyclinE1/CDK2 signaling pathway. (A) Real time PCR detects the expression of *tgfβ1* mRNA fold change of non-fibrosis liver and fibrotic liver for indicated time following ALPPS (n = 4, two-tailed ANOVA). (B) Immunoblotting of lysates prepared from non-fibrosis and liver fibrosis tissues with SMAD2, pSMAD3, SMAD4, SMAD6 antibodies. GAPDH served as a loading control. (C) Western bolt analysis of TGFβ1, pSMAD2, αSMA of liver tissue harvested from DEN treated rats 1, 3,6, 9 and 12 days after DEN withdrawal without ALPPS. GAPDH served as a loading control. This set of data shows that baseline was relatively stable.

Fig S3. Detection of cell cycle related proteins in G1/S phase. (A) Real time PCR detects the expression of ccnd1, ccnd2, ccnd3, ccne1, ccne2, ccne1 at 1st, 2nd, and 3rd day after Step I (n = 4, two-tailed ANOVA). (B) Immunoblotting of lysates prepared from non-fibrosis and liver fibrosis tissues with Cyclin D1, D2, D3, E2, CDK4 and CDK6 antibodies. GAPDH served as a loading control. (C) Western bolt analysis of TGFβ1/pSMAD2/Cyclin E1 pathway in CCl_4-indued fibrotic liver tissues following ALPPS.

Fig S4. Tgfβ1 mRNA fold of DEN-FLR/non-fibrosis FLR was shown at indicated time after ALPPS. This data was calculated based on the data in FigS2a, suggesting that the largest difference in expression of tgfβ1 between non-fibrosis and fibrotic FLRs at day 7.

Fig S5. Immunofluorescence staining demonstrated that TGFβRI presented on the hepatocyte membrane.

Fig S6. P21 is highly expressed in fibrotic liver tissues and decreases with liver proliferation. (A) Immunohistochemical staining using p21 and pSMAD2 antibody in DEN-FLR and AAV-shtgfβ1-DEN-FLR 2 days after ALPPS. CV, Central lobule vein. P21 and pSMAD2 are located far away from the CV area. scale bars, 100um. (B) P21 protein detected by immunoblotting down regulated in regenerative live tissues.

Supplementary Table 1. Primers used in the study.

Primers		Sequences (5'------3')
Cdkn1b	Forward：	CACTGCCGAGATATGGAAGAAGCG
	Reverse：	CCTCTCCACCTCCTGCCACTC
Tgfβ1	Forward：	TGCTTCAGCTCCACAGAGAA
	Reverse：	TCCAGGCTCCAAATGTAGGG
Tgfβr1	Forward：	CCCTCACTAGATCGCCCTTT
	Reverse：	TGCCGATGCTTTCTTGTAGC
Ccnd1	Forward：	ATCAAGTGTGACCCGGACTG
	Reverse：	AGCTTCTTCCTCCACTTCCC
Ccnd2	Forward：	GACCTTCATCGCTCTGTGTGCTAC
	Reverse：	TTGTGCTGCTCTTGACGGAACTG
Ccnd3	Forward：	GAAACCACGCCCCTGACTATTGAG
	Reverse：	AGCAGCCAGGTCCCACTTGAG
Ccne1	Forward：	AAAGAAGAAGGTGGCTCCGA
	Reverse：	ACGCACGCTGAATCATCATC
Ccne2	Forward：	TGTCAAGACGCAGTAGCCGTTTAC
	Reverse：	TCTGGGCTTCTTGTGGAGAGTCTG

Supplementary Table 2. Primary antibodies with indicated concentration for WB, IHC, IF.

Protein	WB	IHC	IF	Specificity	Product code
αSMA	1/1000	1/100		Rabbit	abcam，ab5694
αSMA			1/100	Rabbit	abcam, ab124964
collagen I		1/100		Rabbit	abcam，ab34710
vimentin	1/2000			Rabbit	CST, #5741
TGFβ1	1/1000			Rabbit	abcam，ab179695
TGFβ1			1/50	Mouse	SANTA CRUZ，sc-130348
p-Smad2	1/1000			Rabbit	CST，#18338
p-Smad2		1/20		Rabbit	ZEN Bio,R22952
Smad2	1/1000			Rabbit	CST，#5339
p-Smad3	1/2000			Rabbit	Novus，77836
Smad3	1/1000			Rabbit	CST，#9513
Smad4	1/5000			Rabbit	abcam，ab40759
Smad6	1/100			Rabbit	Novus，NB100-56440
Smad7	1/1000			Rabbit	25840-1-AP，Proteintech
ki67		1/200		Rabbit	abcam，ab16667
GAPDH	1/10000			Rabbit	abcam，ab181602
CDK2	1/1000			Rabbit	CST，#2546
Cyclin D1	1/500			Rabbit	SANTA CRUZ，sc-8396
Cyclin D2	1/1000			Rabbit	CST，#3741
Cyclin D3	1/2000			Mouse	CST，#2936
Cyclin E1	1/1000			Rabbit	CST，#20808
Cyclin E2	1/1000			Rabbit	CST，#4132S
CDK4	1/1000			Rabbit	abcam，ab7955
CDK6	1/1000			Mouse	abcam，ab77674
P21	1/1000			Rabbit	abcam，ab109199
P21		1/20		Rabbit	ZEN BiO, 385235

HNF4α		1/50	Mouse	Abcam，ab41898
HNF4α		1/50	Rabbit	Novusbio，NBP1-89679
GFP		1/100	Mouse	Abcam，ab1218
TGFβR1		1/100	Rabbit	Invitrogen，PA1-38737
p27	1/1000		Rabbit	CST，#3686

Note：CST is the abbreviation of “Cell Signaling Technology”.

Methods

Animals

All animals received humane care according to the "Guide for the Care and Use of Laboratory Animals" and all experimental procedures were conducted under the supervision of the Animal Ethics Committee of Harbin Medical University (HMU), China (No. 2019033). Male Sprague-Dawley rats, 4 weeks of age, were purchased from the Experimental Animal Center of HMU. After arrival, the animals were given ad libitum access to food and water in a vivarium that was maintained at 23 ± 1 °C with a 12/12 h day/night cycle. Animals were randomly divided into groups, with 3–5 animals sacrificed for each data point.

N-diethylnitrosamine (DEN) and carbon tetrachloride (CCL4) administration

DEN and CCL_4 were used to induce liver fibrosis in rats separately. DEN (Sigma Aldrich, 73861, USA) was diluted in sterile phosphate-buffered saline (PBS) and intraperitoneally injected into rats twice a week at a dose of 25 mg/kg from the age of 6 weeks. Sterile PBS was injected into the control group. CCl_4-treated rats were intraperitoneally injected with 40 % CCl_4 olive oil solution (v/v) at 1ml/kg body weight twice a week. The DEN treatment and CCL_4 treatment lasted up to 14 weeks. METAVIR scoring was used to evaluate the degree of liver fibrosis (15).

Adeno-associated virus (AAV) vectors

AAV9 GFAP vectors designed to specifically transfect HSCs in vivo were generated by

Genechem Co. LTD (Shanghai, China). A short hairpin RNA sequence targeting the *TGFβ1* gene was cloned and packaged into the AAV vector (CV258, GFAP-EGFP-MIR155). The vector also encoded a green fluorescent protein (GFP) reporter, allowing for cellular visualization. The TGFβ1 RNA interference sequences used were 5'ACCGCTAGCTAACTGGAGGCTTGCTGAAGGCTGTATGCTG and 3'CAGGACACAAGGCCTGTTACTAGCACTCACATGGAACAAATGGCCCAAGCTTGGT. TTCTCCGAACGTGTCACGT was used as control.

LY2157299 (Galunisertib) administration

LY2157299, a TGFβR1-specific inhibitor, was obtained from Selleck (s2230, USA) and dissolved in 1% sodium carboxymethyl cellulose (CMC) (Selleck, s6703, USA). DEN-treated rats were given either 150 mg/kg galunisertib or 1% CMC intragastrically twice per day for a total of 7 days before the rats were sacrificed (16, 17).

AAV delivery into rat livers

Rats were anesthetized by intraperitoneal injection of 2% pentobarbital. A longitudinal incision was then made in the abdomen to expose the portal vein. 8×10^{11} viral particles of AAV9-GFAP-shTGFβ1 or AAV9-GFAP-shNC in a final volume of 2 mL were injected into the portal vein with a 25-gauge needle, as described previously (18). Four weeks after AAV infection, ALPPS was performed.

Development of the ALPPS rat model

After 4–5 days of DEN/PBS treatment (week 12), rats were weighed and anesthetized by intraperitoneal injection of 2% pentobarbital. A state of deep anesthesia was confirmed by the toe-pinch reflex. Step I: To simulate human ALPPS in rats, portal vein branches were ligated and liver parenchyma was split between the right and left middle lobes. The left lateral lobe (LLL) was resected first, as is often done clinically to remove small colonic liver metastases. Portal branches of the right lobe (RL) and right middle lobes (RML) were individually ligated with 5-0 silk. The portal vein branches and the hepatic artery of the left middle lobe (LML) as well as the caudate lobe (CL), which served as the FLR and represented 26–30% of the total liver volume, were conserved. The liver parenchyma was split between the deportalized RML and the normally perfused LML with bipolar forceps. Step II: Rats underwent a relaparotomy 48 h after Step I to remove the deportalized RL and RML.

Liver sample acquisition

Blood was obtained from the intrahepatic vena cava before organ harvesting and centrifuged at 3000 ×*g* for 30 min. Serum aliquots were collected and stored at -80 °C. Serum alanine aminotransferase (ALT), albumin (ALB), and total bilirubin (TBIL) levels were determined using an automated chemical analyzer (Johnson Vitros 5600 automatic biochemical analyzer, USA). Liver tissues were washed repeatedly with precooled PBS, weighed, and aliquoted. Part of the liver tissue was snap frozen directly and preserved at -80 °C, and part of the tissue later used to extract RNA was placed in a protectant tissue reagent (Qiagen, 76104, Germany) for 24 h at room temperature and

then stored at -80 °C. The remaining liver tissue was fixed in 10% formalin.

Liver regeneration

Liver regeneration was assessed by calculating the ratio of total FLR, including LML and CL, to body weight (FLR/BW). 3–5 animals were used for each time point.

Isolation and culture of primary hepatocytes (PHCs) and HSCs

A two-step collagenase perfusion method was used to isolate and purify rat hepatocytes (19). Briefly, FLR dissected from DEN-treated rats 48 h after ALPPS were perfused with calcium-free Hanks balanced salt solution (HBSS) and then perfused with HBSS containing calcium and collagenase type IV (Gibco, 17104019). Livers were minced and filtered through cotton gauze to liberate the hepatocytes. The hepatocytes were suspended in 7 mL of Earle’s minimum essential medium with 10% fetal bovine serum (FBS), 2% penicillin/streptomycin, 1% glutamine, and 1% nonessential amino acids. The hepatocytes were purified from nonparenchymal cells and nonviable hepatocytes by Percoll density gradient centrifugation at 1000 g for 10 min at 4 °C.

HSCs were isolated from the FLR of DEN-treated or PBS-treated rats 48 h after ALPPS by enzymatic digestion of the liver with collagenase type IV (Gibco, 17104019), Pronase (Roche, 10165921001), and DNase (Sigma, DN25) followed by centrifugation of the crude cell suspension through a density gradient medium (Nycodenz) (20). 6–8 $\times 10^6$ cells from each rat FLR were cultured in Dulbecco's Modified Eagle Media (DMEM) with 10% FBS, 2% penicillin/streptomycin, and 1% glutamine. HSCs and

hepatocytes were cultured at 37 °C in a humidified incubator with 5% CO_2.

BRL-3A culture

The rat hepatic cell line BRL-3A was obtained from National Collection of Authenticated Cell Cultures (Shanghai, China). BRL-3A was cultured in DMEM supplemented with 1% glutamine, 10% FBS, and 1% penicillin/streptomycin at 37 °C in a humidified incubator with 5% CO_2.

Co-culture

PHCs/BRL-3A cells were plated on 10 cm dishes (2×10^5 cells/mL) and incubated at 37 °C in 5% CO_2 for 48–72 h. When the cultures reached ~50% confluence, the medium was replaced with medium used to culture HSCs for 48 h. 10 ng/mL cytokine rat TGFβ1 (rTGFβ1) (Novoprotein, Shanghai, China) and 10 μM galunisertib dissolved in DMSO were added to the medium and the cells were incubated for 24 h.

5-Ethynyl-20-deoxyuridine (EdU) assay

Cells were incubated with EdU (Ribobio, Shanghai, China) for 2 h and processed according to the manufacturer's instructions. After washing with PBS, the cells were treated with 300 μL of Apollo reaction cocktail for 30 min. Then, the DNA contents of the cells in each well were stained with Hoechst. In vivo, 5 mg/kg EdU was injected intraperitoneally 24 h before the animals were sacrificed. The tissues were fixed in 10% neutral formalin, dehydrated, embedded in paraffin, and sectioned at 4 μm. The sections

were incubated with Apollo reagent for 60 min and the nuclei were stained with Hoechst. The cells and tissue sections were visualized under a fluorescence microscope. Quantification of EdU-positive hepatocytes was performed by blinded manual counting of five random visual fields (200×).

Cell Counting Kit-8 (CCK8) assay

CCK-8 assay was used to measure cell viability. Exponentially growing hepatocytes (100 μL, 1×10^5 cells/mL) were seeded on 96-well plates. The plates were then incubated at 37 °C and 5% CO_2 for 48 h. Subsequently, 10 μL of CCK-8 (Beyotime, China) was added to each well. After 2 h, the absorbance at 450 nm was measured using a spectrophotometer.

Quantitative real-time PCR (qRT-PCR)

Total RNA was extracted from 50 mg of tissue using GeneJET RNA Purification Kit (Thermo Scientific, k0732, USA). RNA quality and quantity were assessed using a spectrophotometer. After generation of the complementary DNA sequence (Takara reverse transcription system, rr036, China), qRT-PCR amplification and data analysis were performed on an ABI StepOne Plus Biosystem System using TB Green Premix Ex Taq™ II (Takara, rr820, China). The primers used for qRT-PCR are listed in Supplementary Table 1.

Protein analysis

Total protein was extracted from 50 mg of liver tissue using Tissue Extraction Reagent II (Invitrogen, fnn0081, USA) and an EDTA-free protease inhibitor cocktail kit (Roche, 04693132001). Protein concentration was determined using a BCA protein assay kit (Sangon biotech, c503021, Shanghai, China). Total protein was separated by acrylamide gel and transferred to nitrocellulose. After blocking with 5% bovine serum albumin and incubation with primary and secondary antibodies, blots were displayed on the film and quantified using ImageJ v1.53a (Wayne Rasband, National Institutes of Health, USA). The concentration of TGFβ1 in each sample was measured using an ELISA kit (Sangon biotech, d751002, Shanghai, China).

Immunohistochemistry and picric-sirius red (PSR) staining

Ninety rat liver samples were embedded into a tissue microarray by Outdo Biotech (Shanghai, China), stained with diaminobenzidine (Vector Laboratories, SK-4100), and counterstained with hematoxylin (Thermo Scientific, 7211, USA) to visualize the immunoreaction product following the manufacturers' suggested protocols. Antibodies used are listed in Supplementary Table 2. Liver sections were incubated with PSR solution for 1 h then hematoxylin for 5 min to dye the nuclei. Quantification of Ki67-positive hepatocytes and PSR area were performed by blinded manual counting of five random visual fields (200×).

Immunofluorescence assay

Liver tissue sections or cells were permeabilized with 0.1% Triton X-100 and then

incubated with TGFβ1, α smooth muscle actin (αSMA), hepatocyte nuclear factor α (HNF4α), and GFP primary antibodies. After washing with PBS, the samples were incubated with goat anti-mouse IgG and goat anti-rabbit IgG secondary antibodies. Antifade reagent with the nuclear stain Hoechst (Invitrogen, P36985) was added before imaging. Images were captured on a fluorescence microscope.

Statistical analyses

All statistical analyses were blindly performed. Data are presented as mean ± standard deviation. Differences between groups were assessed by Mann–Whitney U Test. Shapiro–Wilk method was used to test if the data were Gaussian distributed. Two-tailed ANOVA was used for multiple testing and Šidák method was used to justify the data. Statistical analyses were performed using Prism v8.0 (GraphPad, San Diego, CA). $P < 0.05$ was considered statistically significant. Statistical significances are presented as $^*P < 0.05$, $^{**}P < 0.01$, and $^{***}P < 0.001$.
